# ERP Correlates of Simulated Purchase Decisions

**DOI:** 10.3389/fnins.2016.00360

**Published:** 2016-08-08

**Authors:** Patrick D. Gajewski, Jessica Drizinsky, Joachim Zülch, Michael Falkenstein

**Affiliations:** ^1^Leibniz Research Centre for Working Environment and Human Factors at Technische Universität DortmundDortmund, Germany; ^2^LdV-C^3^-Lab, Chair of Industrial Sales Engineering, Faculty of Mechanical Engineering, Ruhr University BochumBochum, Germany; ^3^Institute for Working, Learning and AgingBochum, Germany

**Keywords:** neuroeconomics, decision making, N2, P3b, response-locked P3

## Abstract

Decision making in economic context is an everyday activity but its neuronal correlates are poorly understood. The present study aimed at investigating the electrophysiological brain activity during simulated purchase decisions of technical products for a lower or higher price relative to a mean price estimated in a pilot study. Expectedly, participants mostly decided to buy a product when it was cheap and not to buy when it was expensive. However, in some trials they made counter-conformity decisions to buy a product for a higher than the average price or not to buy it despite an attractive price. These responses took more time and the variability of the response latency was enhanced relative to conformity responses. ERPs showed enhanced conflict related fronto-central N2 during both types of counter-conformity compared to conformity decisions. A reverse pattern was found for the P3a and P3b. The response-locked P3 (r-P3) was larger and the subsequent CNV smaller for counter-conformity than conformity decisions. We assume that counter-conformity decisions elevate the response threshold (larger N2), intensify response evaluation (r-P3) and attenuate the preparation for the next trial (CNV). These effects were discussed in the framework of the functional role of the fronto-parietal cortex in economic decision making.

## Introduction

In daily life we make a lot of decisions. Particularly, purchase decisions are very frequent. Some of them are complex and need a lot of reasoning to be done. This needs time and cognitive resources to compare alternatives and to balance pros and cons. For instance, if we look for a new washing machine we analyze a number of factors before we make a decision: the quality, the label, useful tools, energy consumption, and of course its price. Then, we bring all factors together and decide for product A and not for B. In other words each decision is based on accumulation of evidence until a threshold is reached to select a particular product from two or more alternatives (Brunton et al., 2013). How does the brain do this work? There exists a number of models and empirical studies aiming at explaining the functional mechanisms underlying decision making (Gehring and Willoughby, 2002; Kennerley et al., 2006; Bogacz, 2007; Knutson et al., 2007; Forstmann et al., 2008; Ratcliff and McKoon, 2008; Rangel and Hare, 2010). First, after a potential object of purchase has been perceived and analyzed, memory contents were activated which, are associated with this object. Besides the estimation of its value, each product is also associated with a particular emotional response established in earlier situations, or by experienced reward or punishment (Bechara and Damasio, 2005).

Whereas consolidation of memory units occurs in hippocampus, in the medial temporal lobe, activation, and processing of memory contents proceeds in the prefrontal brain areas (Nyberg et al., 1996; MacLeod et al., 1988; Lepage et al., 2000). The value of a stimulus is evaluated primarily in ventral striatum, particularly in nucleus accumbens (Knutson et al., 2001, 2005, 2007), in the dorsolateral prefrontal cortex, anterior insula (Sanfey et al., 2003), and medial orbitofrontal cortex (Rangel and Hare, 2010), whereas the subthalamic nucleus (STN), a part of basal ganglia, which receive input from medial prefrontal cortex seems to adjust the decision threshold by accumulation of information (Keuken et al., 2015; Herz et al., 2016). More important in the present context, however, is the anterior cingulate cortex (ACC) that is strongly interconnected with prefrontal cortex, hypothalamus, amygdala, and basal ganglia like STN. Moreover, ACC plays an integrative role between cognitive contents, and emotional responses (Paus, 2001). In addition it has been shown that ACC is crucial for error monitoring (Ullsperger and von Cramon, 2004) and action based choices (Kennerley et al., 2006; Rudebeck et al., 2008). This area is additionally involved in detection and resolution of cognitive conflicts (van Veen and Carter, 2002; Botvinick et al., 2004; Botvinick, 2007) in order to select the appropriate response in two alternative choices (Botvinick, 1999; Turken and Swick, 1999). Increased activity in ACC correlates with the magnitude of anticipated consequences of actions (Sanfey et al., 2006) and adjusts the distance to the decision threshold (Domenech and Dreher, 2010; Keuken et al., 2015). Moreover, increased dorsal ACC activity was strongly associated with choice difficulty, as it is more active during overriding the default alternative (Shenhav et al., 2014). In other words, ACC seems to act as an instance that integrates and accumulates evidence from different subcortical and cortical sources (d'Acremont et al., 2013) and resolves conflicting response tendencies to reach the threshold and complete a decision, similar to the proposed models of decision making (Bogacz, 2007; Keuken et al., 2015; Herz et al., 2016). There is also ample evidence that emotions play a crucial role in economic decision making (Kahneman et al., 1999; Shiv and Fedorikhin, 1999; Loewenstein and Lerner, 2003; Bernheim and Rangel, 2004; Steffen et al., 2009; Zhao et al., 2015), and deficits in emotional processing can impair the quality of decision making (Bechara and Damasio, 2005). Thus, the close co-location of cognitive and affective subareas of the ACC suggests an intensive interaction between cognition and emotion involved in decision processes (Bush et al., 2000).

To investigate how brain activity is linked to decision making, event-related potentials (ERPs) can be applied which offer an excellent time resolution allowing investigation of discrete processing steps. Thus, ERPs may be suitable to analyze the processing steps involved in purchase decisions. However, purchase decision studies using ERPs are scarce.

In a study conducted by Chen et al. (2010b) participants had to decide whether to buy a book in an online shop given the quantity of positive and negative reviews of the book. Two types of decisions were distinguished: Conformity decisions were made if the participant decided to buy a book on the basis of positive reviews or not to buy it for negative reviews. Counter-conformity decisions were made if the participant acted contrary. Reaction time (RT) for counter-conformity choices was longer than for conformity choices. Moreover, between 300 and 600 ms after stimulus-onset, a long-lasting negative potential (N500) occurred when participants made a counter-conformity decision. This indicates that counter-conformity decisions are associated with conflicts which need more time for resolving and show up in a deflection of the N500. Regarding positive potentials like P3b, Chen et al. (2010a,b) reported that the P3b associated with allocation of cognitive resources (Polich, 2007) was mostly pronounced during conformity decisions, whereas counter-conformity decisions elicited a much lower P3b.

In another study by Steffen et al. (2009) pictures of an apartment were presented to the participants who had to decide whether to rent the apartment or not. The decision should be based on the price or on the brightness of the apartment. Additionally, in two-thirds of the experiment, happy, and unhappy faces (emotional primes) were placed right before the picture of the apartment was presented. Irrespective of affective expression, primes accelerated decisions. This suggests that faces with different emotional expressions can act as primes influencing decision making. Furthermore, decisions based on the price of the apartment were made faster and evoked larger N200 than decisions based on brightness. Thus, the N200 amplitude seems to reflect a sensitive measure for the interplay between cognitive and affective aspects of a decision.

The studies by Chen et al. (2010b) and Steffen et al. (2009) highlighted effects of negative components N500 and N200, correspondingly which may be related to decision making. The differences in latency may stem from different design of the tasks and decision complexity. This component, known also in the literature as the fronto-central N2 has been linked to key task related processes: detection of mismatch and response monitoring (Folstein and Van Petten, 2008; Huster et al., 2013; Hämmerer et al., 2014, for reviews), conflict processing (van Veen and Carter, 2002; Yeung et al., 2004; Yeung and Cohen, 2006), stimulus classification and decision making (Ritter et al., 1979, 1982), and response selection (Gajewski et al., 2008, 2010, 2011; Gajewski and Falkenstein, 2012). In case of response conflict the selection of the correct response is more demanding as indicated by the larger and often delayed N2. As the N2 is generated in ACC (Yeung and Cohen, 2006) or more exactly at the border between ACC and pre-supplementary motor area (pre-SMA; Ullsperger and von Cramon, 2004) known to integrate inputs from different brain areas to enable motor performance (Nachev et al., 2008) and decision making (Forstmann et al., 2008; Rangel and Hare, 2010; Keuken et al., 2015), this potential seems to reflect analysis and resolution of conflicts between contradictory cognitive contents to select and elicit a unique response. Taking together, the fact that ACC—Pre-SMA compound enables convergent processing, i.e., comparison of different alternatives in order to select one of them and to translate in an appropriate action, qualifies this neuronal system to make decisions (Sanfey et al., 2006; Botvinick, 2007; Forstmann et al., 2008; Bogacz et al., 2010; Keuken et al., 2015). Furthermore, the P3 has also been related to decision or post-decision processes (Squires et al., 1977; Finnigan et al., 2002; Nieuwenhuis et al., 2005; Verleger et al., 2005). Nieuwenhuis et al. (2005) suggested that the phasic response of the locus coeruleus underlying the P3b is driven by the activity of units representing the winning alternative of the decision process and is elicited as soon as this crosses the decision threshold. Verleger et al. (2005) proposed that “decision” implies a direct link from the percept to the alternatives to be decided upon, which are the responses and concluded that P3b should be equally related both to stimulus and response. Indeed, in their study Verleger and colleagues found the same P3b amplitude in stimulus- and response-locked P3b, concluding that the P3b reflects a consequence of a decision rather than the process of deciding itself, corroborating the proposal of Nieuwenhuis et al. (2005). In the stimulus-locked averages the P3b is often smeared out due to its time-relation to the response (e.g., Falkenstein et al., 1994). In case of overt responses the P3b can be measured in the response-locked averages, where it shows up as a well-defined positivity shortly after the response (Friedman et al., 1978; Verleger et al., 2005; Gajewski and Falkenstein, 2011).

The aim of the present study is to investigate the neuronal underpinnings of decision making in an economic context. In particular, participants were presented a face with a positive (friendly), neutral (emotionless), or negative (angry) expression in order to induce a positive, neutral, or negative emotion assumed to enhance or to reduce the willingness to buy a product and/or to accelerate the decision (Bechara and Damasio, 2005; Steffen et al., 2009). Afterwards, a photo of a technical product was displayed. The trial was terminated by presenting a price and the participants had to make a fast decision to purchase this product or not. Each product was combined with low or high prices. The main output measures were reaction times, variability of speed, and most importantly, the ratio of buying a product or not as a function of high and low prices. In contrast to the studies by Chen et al. (2010a,b) in which participants decided to buy a book based on positive or negative book reviews provided by others, in the present study the decision was based on the subjective estimation whether the price of a product is appropriate or not. We use here the same terminology as Chen et al. (2010a,b) but defined conformity and counter-conformity decisions slightly different. Conformity decisions were made when participants decided to buy a product that was cheaper than the mean price estimated in the pilot study or not to buy a product that was more expensive than the mean estimated price of this product. Counter-conformity decisions denote situations when the participants do the opposite, i.e., buy an expensive and not buy a cheap product.

The specific hypothesis was that a higher cognitive conflict would be produced in counter-conformity decisions when participants decided to buy an expensive product or not to buy a cheap product, whereas no conflict should be evoked in conformity decisions. It should be noted that the term conflict does not correspond to the classic “response conflict” indicating a conflict between incompatible response tendencies (e.g., Yeung and Cohen, 2006). In the present paradigm there is no correct or false response. Instead, a conflict between cognitive contents may also arise when one decides to do or not to do something against universal expectations or beliefs.

Generally, we expect that cheap products are bought more frequently than expensive products and the decision time should be longer and the variability of speed larger in counter-conformity decisions. According to existing literature we expect that emotional face expression should affect the rate of purchase decisions (e.g., Steffen et al., 2009). The ERPs should shed more light on the processing of such decisions. According to the conflict hypothesis of the N2, we predicted that the N2 associated with response selection (i.e., a binary decision making) should be larger and/or delayed in counter-conformity than conform purchase decisions. Moreover, we investigated the P3 complex to assess effects on post-decision processes, which are probably reflected in the P3b (Nieuwenhuis et al., 2005; Verleger et al., 2005). Because of a possible overlap of the N2 and subsequent negativities with the P3 complex we also analyzed the response locked P3 (r-P3) relative to a pre-response baseline. By this technique the influence of pre-response negativities such as the N2 should be removed and true effects on the P3b unveiled.

## Methods

### Participants

Forty-two participants (27 males) aged from 20 to 29 (mean ± SD: 23.5 ± 2.3 years) were included in the study. Participants were recruited through several flyers that were distributed through university mailing lists or placed on the university campus. The sample consisted of engineering science students (66.6% Sales Engineering and Product Management). Thirty-eight participants were right-handed and all had normal or corrected-to-normal vision. They were paid (25 €) for their participation. The study was approved by the institutional review board. Written consent was obtained according to the Declaration of Helsinki.

### Stimuli and tasks

**Figure 1:**
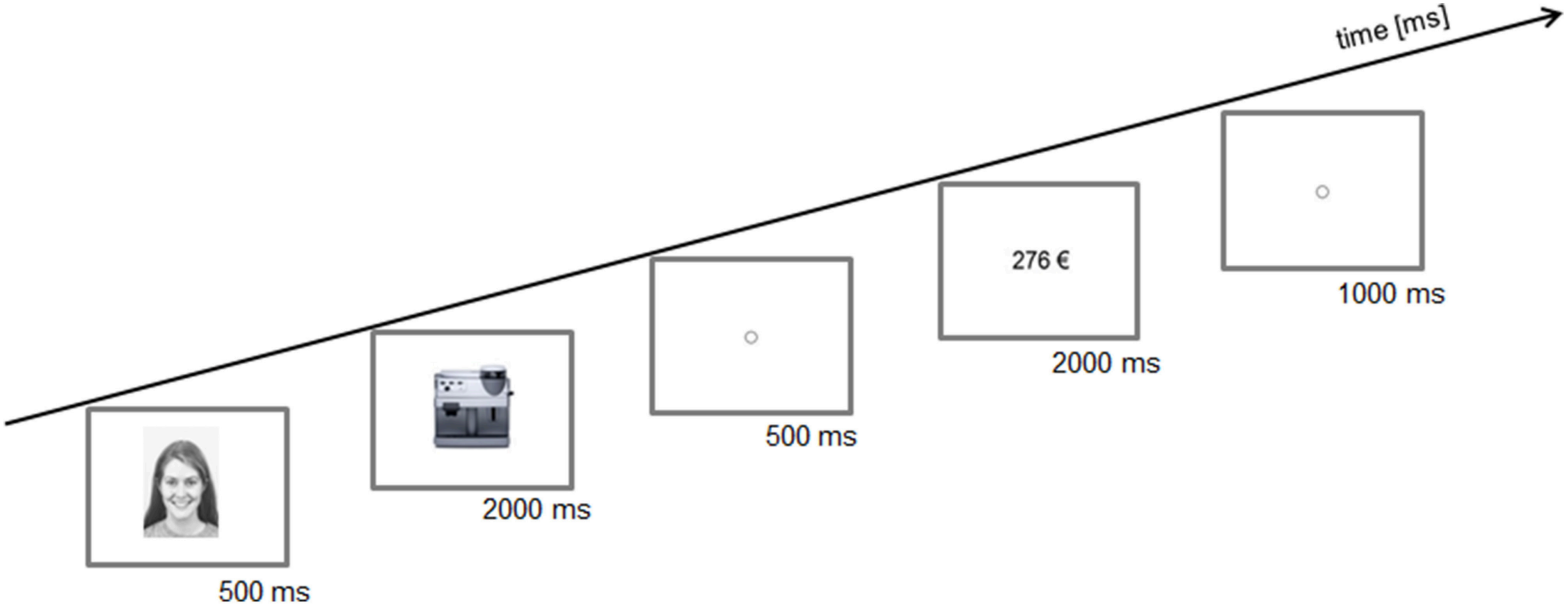
**Schematic illustration of a trial**. First a positive, neutral, or negative face was presented. Then, a product was offered and afterwards a price was presented. The participants should decide to buy or not to buy the product for the given price. Presentation times of the images in milliseconds (ms).

Figure 1 shows a schematic illustration of a trial.

At the beginning of each trial one of three possible facial expressions (positive, neutral, or negative) was shown in the center of the monitor for 500 ms. Images of 45 individuals (23 males) were taken from Karolinska Directed Emotional Faces (Lundqvist et al., 1998), were adjusted to a size of 562 × 762 pixels and converted to black and white. Then 1 of 90 images of technical products (e.g., a coffee maker, camera, washing machine etc.,) appeared in the center of the monitor for 2000 ms. Images of the products were bought from the online image database (Fotolia, 2012) and adjusted to a width of 562 pixels. The height was adjusted automatically according to the given relations of the images. After the product image disappeared, a fixation point was shown for 500 ms. Then the price of the product appeared in the center of the monitor. The prices were displayed in Euro (e.g., 276 €). They were written in black Arial font.

The prices of the products were determined in a pilot study. They were presented to 39 students (14 males) to estimate the average price for each product. The average prices ranged from 2 to 973 Euro (see Table 1). Based on those prices, a set of seven prices per product was calculated (−60, −40, −20%, average price, +20, +40, +60%).

**Table 1:** **List of products used in the study**.

**Product**	**Mean**	**SE**	**SD**	**Min**	**Max**	**−60%**	**−40%**	**−20%**	**+20%**	**+40%**	**+60%**	**Low**	**High**
Air conditioning	227	40	248	25	1500	91	136	182	273	318	364	136	318
Fridge	589	49	308	100	1800	236	354	471	707	825	943	354	825
Laptop	894	92	573	80	3000	358	536	715	1073	1251	1430	536	1251
Treadmill	973	158	988	70	3800	389	584	778	1167	1362	1556	584	1362
PC Mouse	17	2	11	3	50	7	10	14	20	24	27	10	24
Microwave old	73	11	69	25	450	29	44	58	87	102	116	44	102
Microwave new	120	13	80	40	350	48	72	96	145	169	193	72	169
Mixer	41	2	15	20	75	16	25	33	49	58	66	25	58
Chainsaw	177	17	108	30	550	71	106	141	212	247	283	106	247
Sewing machine	129	11	69	20	350	52	78	104	155	181	207	78	181
Navigation device	108	6	40	25	199	43	65	86	129	151	172	65	151
Pizza oven	96	16	98	19	500	39	58	77	116	135	154	58	135
Record player	102	14	85	20	500	41	61	81	122	142	163	61	142
Hand-held blender	34	3	18	10	80	13	20	27	40	47	54	20	47
Lawnmower	219	15	95	60	450	87	131	175	262	306	350	131	306
Electric shaver	78	5	33	25	150	31	47	63	94	110	125	47	110
Multiple plug	13	2	10	2	60	5	8	10	15	18	20	8	18
Scanner	60	5	31	7	150	24	36	48	72	85	97	36	85
Vacuum cleaner	120	12	75	40	400	48	72	96	144	168	192	72	168
Floor lamp	47	3	22	20	100	19	28	38	57	66	75	28	66
Stereo system	114	9	53	25	299	45	68	91	136	159	182	68	159
Tablet	291	34	211	10	750	117	175	233	350	408	466	175	408
Flashlight	12	1	8	2	50	5	7	9	14	16	19	7	16
Keyboard	33	3	19	5	80	13	20	26	39	46	52	20	46
Toaster	25	2	11	10	50	10	15	20	30	35	40	15	35
Security camera	200	34	212	10	1000	80	120	160	240	280	320	120	280
USB stick	11	1	5	5	25	4	7	9	13	16	18	7	16
Ventilator	40	3	19	15	90	16	24	32	48	56	64	24	56
Multi socket	10	1	4	2	20	4	6	8	12	14	16	6	14
Video camera	511	99	619	30	3500	204	306	409	613	715	817	306	715
Digital kitchen scale	27	3	18	8	90	11	16	21	32	37	43	16	37
Washing machine	390	42	264	25	1000	156	234	312	468	546	624	234	546
Water tap	120	25	158	15	600	48	72	96	144	168	192	72	168
Electric kettle	25	2	15	8	70	10	15	20	30	35	40	15	35
Level	15	1	7	3	30	6	9	12	18	21	24	9	21
Waffle iron	26	2	12	5	60	10	16	21	31	37	42	16	37
Cordless drill	46	4	22	15	100	18	28	37	55	64	74	28	64
Batteries	8	1	4	2	18	3	5	6	9	11	12	5	11
Flat screen TV	636	182	1134	100	7000	254	382	509	763	890	1018	382	890
Cordless screwdriver	100	13	81	30	500	40	60	80	120	140	160	60	140
Iron	46	3	19	10	100	18	28	37	55	64	73	28	64
DVD player	76	8	51	30	250	30	46	61	91	107	122	46	107
Ceiling lamp	138	16	102	25	500	55	83	111	166	194	221	83	194
Voice recorder	42	4	23	10	100	17	25	33	50	58	67	25	58
Grinder	45	3	20	15	90	18	27	36	54	63	72	27	63
Printer	126	13	79	30	399	50	76	101	151	177	202	76	177
Egg cooker	21	2	14	5	90	8	13	17	25	29	34	13	29
Electric toothbrush	38	4	22	9	100	15	23	31	46	54	61	23	54
Fax machine	95	8	47	20	230	38	57	76	114	132	151	57	132
HDD	69	5	30	10	150	28	42	55	83	97	111	42	97
Hairdryer	29	2	12	10	60	11	17	23	34	40	46	17	40
Garden hose	64	6	38	15	150	25	38	51	76	89	102	38	89
Dishwasher	398	23	146	100	699	159	239	318	477	557	636	239	557
Light bulb	4	0	3	1	15	2	2	3	5	5	6	2	5
Barbecue grill	45	4	25	10	120	18	27	36	54	63	72	27	63
Hand mixer	28	2	12	10	75	11	17	22	33	39	44	17	39
Electric hedge clipper	87	7	44	20	250	35	52	69	104	121	139	52	121
Radiator	245	47	294	50	1780	98	147	196	294	343	392	147	343
Range with oven	356	31	192	50	900	142	214	285	427	499	570	214	499
Extension cord reel	38	4	26	10	120	15	23	31	46	54	61	23	54
Coffee machine	57	5	32	7	165	23	34	46	69	80	92	34	80
Espresso machine	276	35	221	30	1000	110	166	221	331	386	442	166	386
Electric coffee mill	165	32	200	14	800	66	99	132	197	230	263	99	230
Wall lamp	47	4	24	10	100	19	28	37	56	66	75	28	66
Phone	53	5	34	5	150	21	32	43	64	75	85	32	75
Portable DVD Player	102	10	64	15	300	41	61	81	122	143	163	61	143
Clamp	14	1	6	3	30	6	9	11	17	20	23	9	20
Projector	326	27	169	50	899	130	195	261	391	456	521	195	456
SD card	13	2	11	1	70	5	8	10	15	18	21	8	18
Home camera	346	63	391	46	2000	138	208	277	415	485	554	208	485
Headphones	57	7	44	8	250	23	34	45	68	79	91	34	79
Digital reflex camera	535	66	412	70	2500	214	321	428	642	749	856	321	749
Headset	37	8	48	5	299	15	22	29	44	51	59	22	51
Sheet sander	74	9	54	15	300	29	44	59	88	103	118	44	103
Jig saw	85	10	60	20	350	34	51	68	102	119	136	51	119
Digital camcorder	121	13	81	30	400	48	73	97	145	170	194	73	170
Remote control	17	1	9	2	45	7	10	14	20	24	27	10	24
Radio	48	6	36	5	150	19	29	38	57	67	76	29	67
Polishing machine	301	32	200	20	900	121	181	241	362	422	482	181	422
Digital scale	34	5	32	10	202	13	20	27	40	47	54	20	47
Paper trimmer	32	2	15	10	70	13	19	26	39	45	51	19	45
Telescope	263	49	305	40	1200	105	158	211	316	369	421	158	369
Smoke detector	21	3	21	3	120	9	13	17	26	30	34	13	30
Blood pressure meter	90	13	84	15	500	36	54	72	108	126	144	54	126
Ironing board	33	3	16	15	85	13	20	26	39	46	52	20	46
Curling iron	30	3	18	8	90	12	18	24	36	42	48	18	42
PC monitor	249	27	169	50	900	100	149	199	299	349	398	149	349
Speakers	696	206	1288	60	8000	278	418	557	835	974	1114	418	974
Wrench	12	1	7	3	35	5	7	9	14	16	19	7	16
Window	305	59	367	50	2000	122	183	244	366	427	488	183	427
Smartphone	499	27	168	100	750	200	300	400	599	699	799	300	699
Old cell phone	198	19	120	10	500	79	119	158	237	277	316	119	277
Hair straightener	39	5	32	13	200	16	24	31	47	55	63	24	55
Blue ray player	71	7	46	25	200	29	43	57	86	100	114	43	100
Recordable disc	2	1	4	0	20	1	1	2	3	3	4	1	3
MP3 Player	57	6	36	15	150	23	34	46	68	80	91	34	80
Mean	146	20	124	23	643	59	88	117	176	205	234	88	205

The product prices were evaluated in the pilot study by an independent sample of 39 students. The table presents the mean estimated price, standard error (SE), standard deviation (SD), minimum (min), and maximum (max) price. −60, −40, −20, +20, +40, and +60% represent the price levels, which were pooled for the two final price categories “low” and “high.” Mean values of each parameter were included in the bottom of the table. See also method section for further information.

Thus, each link of product and facial expression was presented once with each of the seven price levels. One of the three facial expressions of 10 different individuals was presented in 10 consecutive trials to reach a stable effect of face expression. The order of the three face expressions was counterbalanced between participants. Products and the corresponding price levels were pseudorandomized and the restricting factor was face expression. Participants performed 3 blocks of 210 trials (630 trials in total). Each block of 210 trials included 30 objects with the corresponding 7 prices, linked to 15 different faces with 3 face expressions.

Prices were presented up to 2000 ms unless the response button was pressed. When no response occurred, the trail was categorized as a miss. The participants were asked to press a key to indicate whether or not they want to purchase the product for the presented price. The yes/no mapping for the response keys was counterbalanced between the subjects. Before the next trial started, a fixation point was displayed for 1000 ms. All stimuli were presented on a white background. The whole experiment took about 60 min. The experiment was programmed with E-Prime 2.0 (Psychology Software Tool, INC.).

### ERP recording

EEG was recorded from 32 scalp electrodes and included 8 midline electrodes and 12 electrodes on each hemisphere positioned according to the 10–20 system (Jasper, 1958). The vertical EOG and horizontal EOG were recorded from electrodes placed supra- and infra-orbital to both eyes and lateral to the outer canthi of both eyes. Electrodes were re-referenced offline to the linked mastoids. EEG and EOG were sampled continuously with a rate of 1000 Hz. ERPs were filtered digitally offline by a high (0.05 Hz) and low pass filter (17 Hz). Electrode impedance was kept below 10 kΩ. The amplifier bandpass was 0.01–140 Hz. Amplitudes that exceeded ±150 μV were rejected. Artifacts of eye movements were corrected by the ocular correction algorithm of Gratton et al. (1983).

### Data analysis

As the facial expression did not reveal any effects or interactions in the behavioral and ERP data, the data were pooled across the three emotional expressions. Moreover, to simplify the data analysis and to increase the number of trials per condition for the ERP analysis, the upper price categories (+20, +40, +60%) were pooled and formed the high price category. Similarly, the lower price categories (−20, −40, −60%) formed the low price category. Analyses of variance (ANOVAs) were performed for the purchase rate, the mean reaction time (RT) and individual standard deviations (ISD) of RTs and the ERP parameters. For the RT analysis the first trial of each block, trials with RTs faster than 100 ms and slower than 2000 ms were excluded from further analysis. The ISD analysis was performed to investigate the individual speed variability between the conditions. The analyses included the within-subject-factors Price (high, low), and Decision (purchase, no purchase). The purchase rate was analyzed as a function of Price (high, low). All *F* ratios associated with repeated measures were assessed using degrees of freedom corrected with the Greenhouse-Geisser procedure. ERPs were analyzed by Vision Analyzer 2.0 (Brain Products, Munich, Germany). Stimulus-locked ERPs were computed beginning 200 ms before and ending 2000 ms after price onset. Response-locked ERPs were computed beginning 500 ms before and ending 2000 ms after response onset. The N2 was analyzed as the peak amplitude in a 200–450 ms time window after price-onset at Fz. The P3a at Fz, and P3b at Pz were analyzed as the peak amplitude in a 300–600 ms time window after price onset. Furthermore, the response-locked P3 (r-P3) was quantified as the peak amplitude in a 50–250 ms time window after the response at Cz where it was maximum. Finally, later post-response ERPs were investigated as after visual inspection of the grand averages large differences between conditions were apparent. Thus, a response-locked negative ERP was analyzed as the peak amplitude in a 200–600 ms time window post-response at Pz. The electrodes were selected according to the topographical distribution of the components illustrated in **Figures 3**, **5**.

In case of significant interactions, comparisons were performed using *post-hoc* tests.

## Results

### Behavioral data

#### Purchase rates

**Figure 2:**
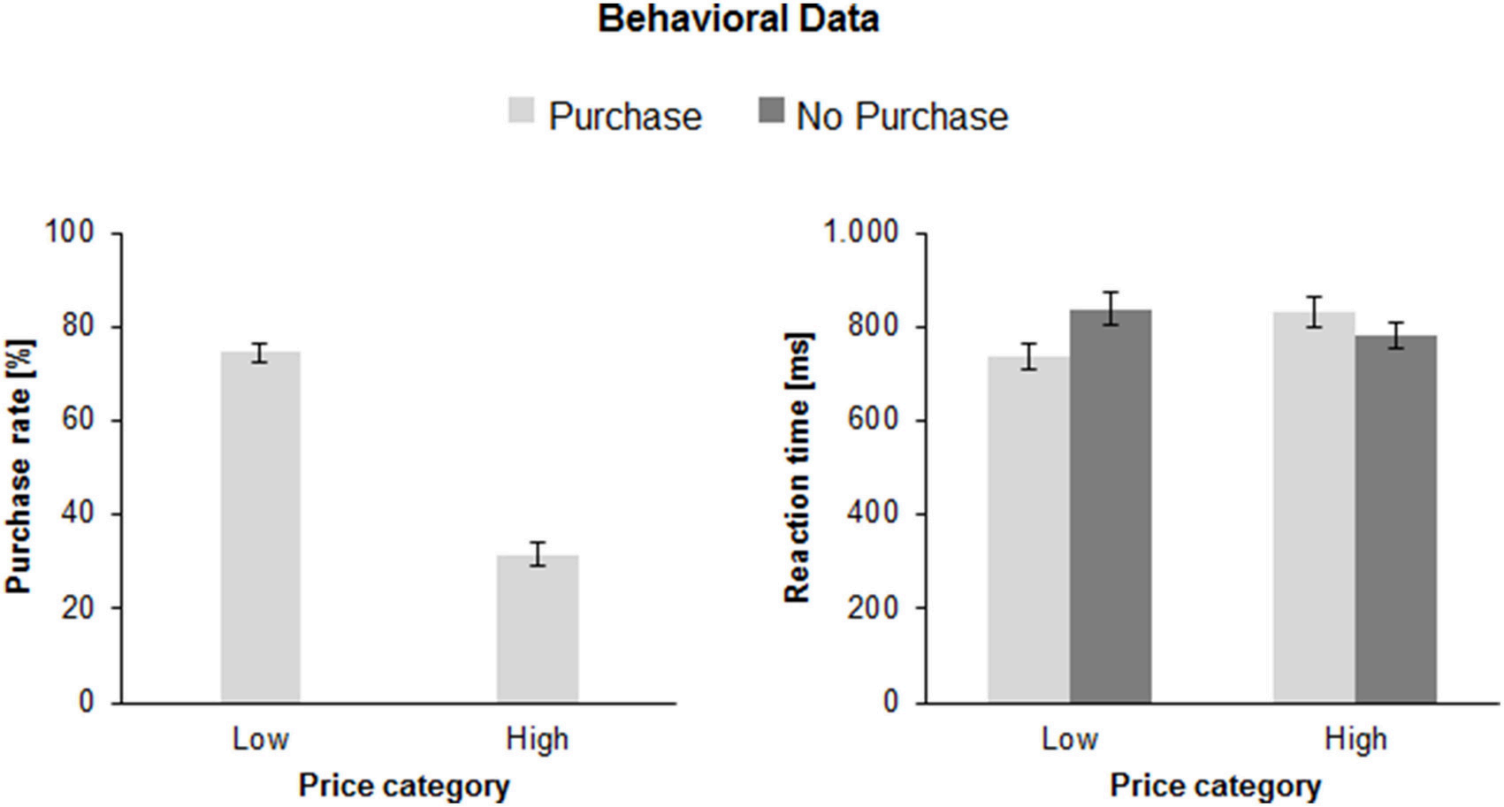
**Mean purchase rates (left) for low and high price category products in percent (%) and mean reaction times (right) as a function of Price Category (low, high), and Purchase Decision (purchase, no purchase) in milliseconds (ms)**. The error bars reflect standard deviations (SDs).

The ANOVA of the purchase rates revealed significantly more purchase decisions for low prices (74.5 ± 2.0%) than for high prices [31.4 ± 2.5%; *F*_(1, 41)_ = 173.9, *p* < 0.0001, η^2^ = 0.81]. There was no effect of face expression or interaction Face Expression × Price (both *F*'s < 1). Figure 2 (left) illustrates the mean purchase rates pooled for all three face expressions.

#### Reaction times

To ensure that RT data are normally distributed we conducted the Kolmogorov–Smirnov test for one sample. This test revealed *p* = 0.2, indicating no significant difference between the present RT- and normal-distribution and consequently no violation of normality assumption. In order to confirm this result with a more sensitive test we conducted the Shapiro–Wilk test again for all 12 RT-variables. The *p*-values varied between *p* = 0.3 and *p* = 0.07, corroborating the normality assumption of the RT-data. The Mauchly's test of sphericity was far from significance (all χ^2^'s < 1), indicating that sphericity was not violated in the present study.

The ANOVA revealed a main effect of Price [*F*_(1, 41)_ = 11.8, *p* < 0.01, η^2^ = 0.22]: the RT for high prices was longer (806.3 ± 28.3 ms) than for low prices (786.4 ± 28.8 ms). Importantly, there was an interaction between Price and Purchase Decision [*F*_(1, 41)_ = 51.3, *p* < 0.001, η^2^ = 0.56], indicating longer RT when participants decided to purchase an expensive product [830.7 ± 30.2 ms; *F*_(1, 41)_ = 8.1, *p* < 0.01, η^2^ = 0.17] than when they decided not to purchase (781.9 ± 29.0 ms). In contrast, when the price was low, the RTs were longer when participants decided not to purchase (836.9 ± 33.9 ms) as when they decided to purchase [735.8 ± 25.9 ms; *F*_(1, 41)_ = 32.4, *p* < 0.001, η^2^ = 0.44]. Figure 2 (right) illustrates this interaction. There was no effect or interaction with facial expression (all *F*'s < 1). Exactly the same pattern of results was obtained when median RTs were considered as dependent variable or if the trials with RTs longer than mean RT + 2SDs (instead of 2000 ms cut-off) were excluded from the analysis.

#### RT variability

An interaction between Price and Decision [*F*_(1, 41)_ = 31.7, *p* < 0.001, η^2^ = 0.44] showed that in case of high prices the ISDs of mean RTs were higher for positive (311.3 ± 10.7 ms) than for negative purchase decision [281.2 ± 9.9 ms; *F*_(1, 41)_ = 23.1, *p* < 0.001, η^2^ = 0.36]. In contrast, in case of low prices, the ISDs were higher for negative (304.9 ± 11.7 ms) than for positive decisions [273.7 ± 9.6 ms; *F*_(1, 41)_ = 13.8, *p* < 0.01, η^2^ = 0.25]. No effects or interactions with facial expressions were found (all *F*'s < 1).

### ERP data

#### Target-locked ERPs

There was no effect of the facial expression. Hence, all data were pooled across the three types of facial expression.

**Figure 3:**
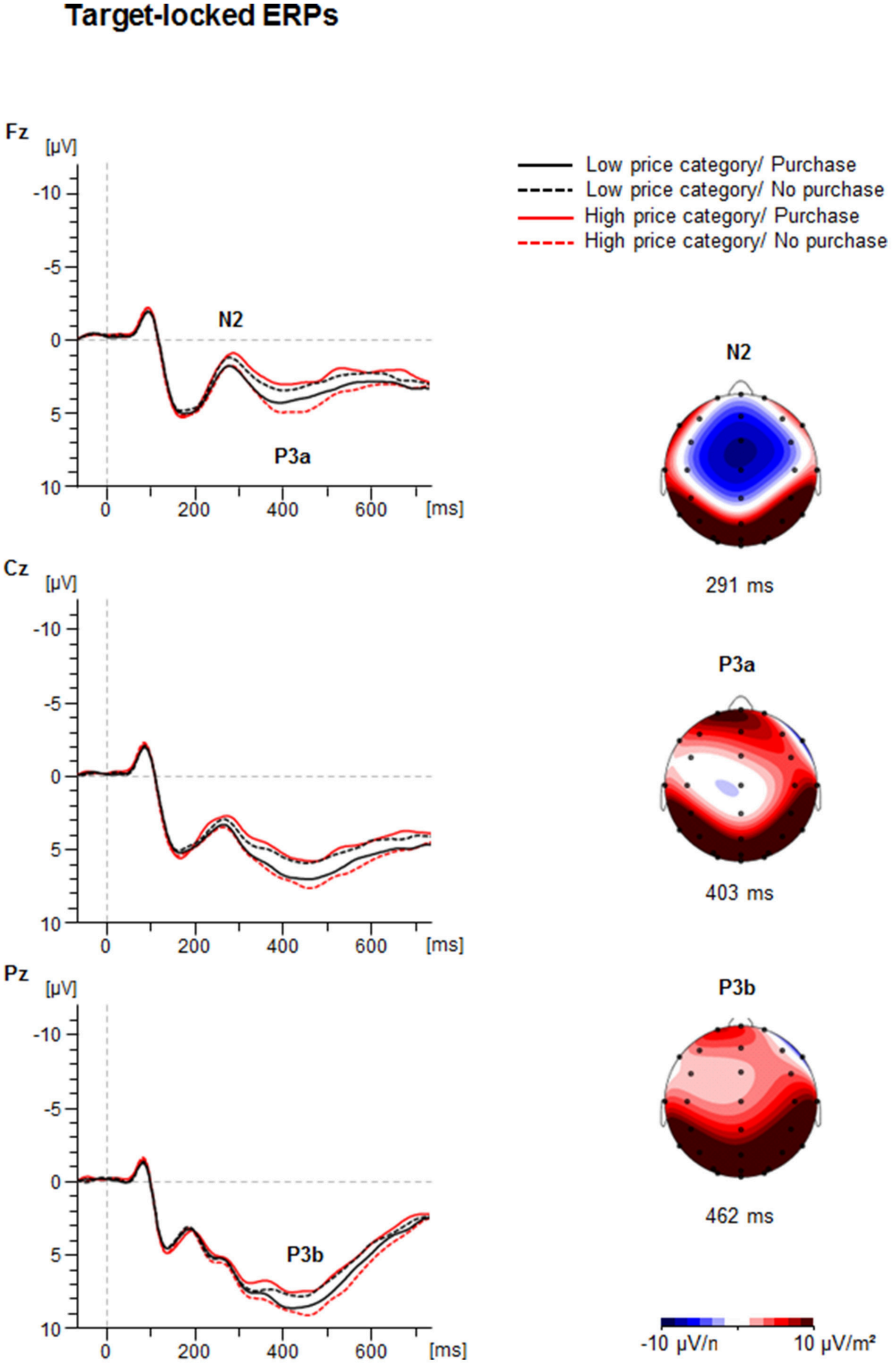
**Target-locked ERP-waveforms as a function of Price Category (low, high) and Purchase Decision (purchase, no purchase) at electrodes Fz, Cz, and Pz**. Dashed vertical line indicate price-onset (0 ms). The Current Source Density maps (CSDs) illustrate a mean activity distribution for the N2 at 291 ms, for the P3a at 403 ms, and for the P3b at 462 ms. Note, the amplitude of the grand average does not necessarily match the amplitude of the peak amplitudes presented in the diagrams (c.f. Figure 4). The generally lower amplitude values in the grand average are due to individual latency jitter of components which reduce its amplitude after grand averaging relative to the peak analysis used for statistics. In contrast, the amplitude diagrams represent the mean of individual maximal or minimal peak amplitudes at the individual latency.

Figure 3 illustrates the grand average of the target-locked ERP-waveforms and the Current Source Density Maps (CSDs), showing the mean activity distribution of the analyzed ERPs. The N2 was seen as a negative peak with frontocentral maximum at about 280 ms, P3 was seen as a positive peak with different latencies with frontal (P3a) and parietal (P3b) maximum.

##### N2

**Figure 4:**
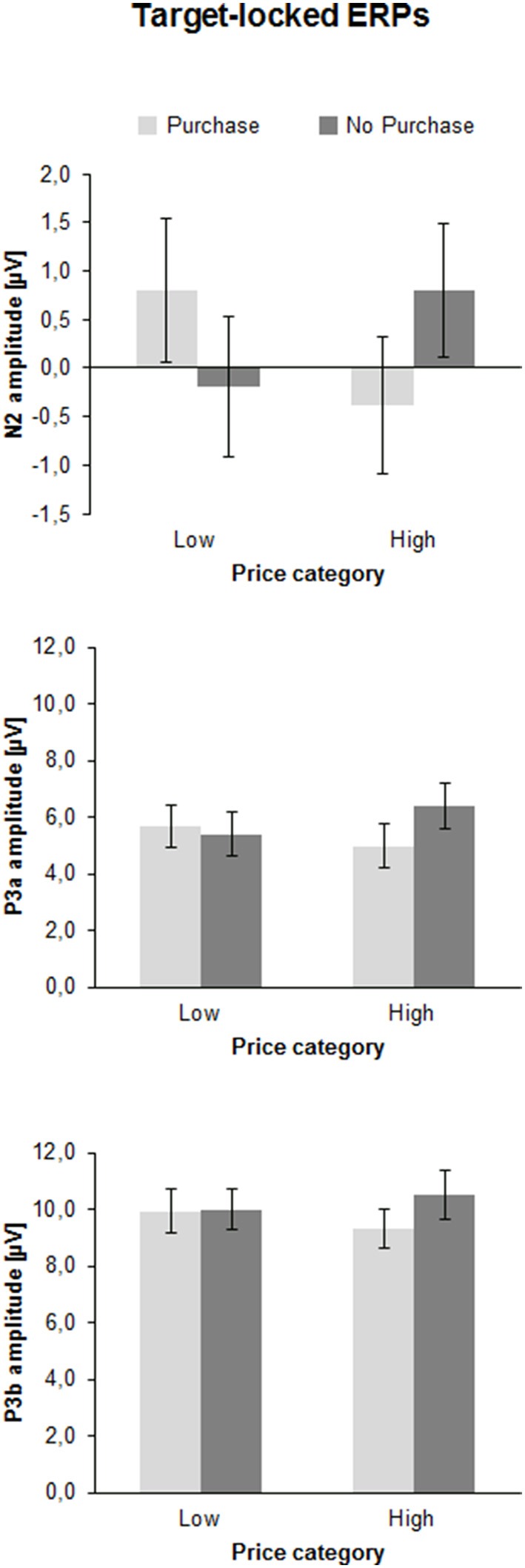
**Mean peak amplitudes of the N2, P3a, and P3b in microvolts (μV) of the price-locked ERPs as a function of Price Category (low, high), and Purchase Decision (purchase, no purchase)**. The error bars reflect SDs.

The ANOVA revealed a significant interaction between Price and Decision illustrated in Figure 4 [*F*_(1, 41)_ = 22.1, *p* < 0.001, η^2^ = 0.35]. In case of high prices a more negative N2 occurred when participants decided to purchase (−0.4 ± 0.7 μV) vs. when they decided not to purchase [0.8 ± 0.7 μV; *F*_(1, 41)_ = 17.6, *p* < 0.001, η^2^ = 0.30]. In contrast, in case of low prices the N2 was more negative when participants decided not to purchase (−0.2 ± 0.7 μV) compared to purchase [0.8 ± 0.7 μV; *F*_(1, 41)_ = 6.9, *p* < 0.05, η^2^ = 0.14].

The N2 latency revealed a main effect of Decision [*F*_(1, 41)_ = 4.3, *p* < 0.05, η^2^ = 0.10], suggesting shorter N2 latencies when participants decided to purchase (294.2 ± 6.9 ms) compared not to purchase (304.5 ± 6.6 ms).

##### P3a

The ANOVA of the P3a amplitude showed a significant interaction between Price and Decision [*F*_(1, 41)_ = 9.2, *p* < 0.01, η^2^ = 0.18], as shown in Figure 4. For high prices, the P3a was larger when participants decided not to purchase (6.4 ± 0.8 μV) than when they decided to purchase [5.0 ± 0.8 μV; *F*_(1, 41)_ = 14.2, *p* < 0.01, η^2^ = 0.26]. For low prices the P3a did not differ between the positive and negative decision (5.7 ± 0.8 μV vs. 5.4 ± 0.8 μV, *p* > 0.10). No latency effects were obtained.

##### P3b

The ANOVA of the P3b peak amplitude yielded an effect of Decision [*F*_(1, 41)_ = 6.0, *p* < 0.05, η^2^ = 0.13], indicating a larger P3b for negative (10.2 ± 0.8 μV) than positive choices (9.6 ± 0.7 μV). Furthermore, there was an interaction between Price and Decision [*F*_(1, 41)_ = 4.8, *p* < 0.05, η^2^ = 0.11]. For high prices the P3b was larger for non-purchase (10.5 ± 0.8 μV), than for purchase decisions [9.3 ± 0.7 μV; *F*_(1, 41)_ = 10.3, *p* < 0.01, η^2^ = 0.20], whereas for low prices, no difference between the decision for purchase (9.9 ± 0.8 μV) and the decision not to purchase was found (10.0 ± 0.7 μV, *p* > 0.10).

P3b latency also showed an interaction between Price and Decision, [*F*_(1, 41)_ = 4.6, *p* < 0.05, η^2^ = 0.10]. However, no significant differences were found between positive and negative decision either for high prices (412.9 ± 11.7 vs. 424.9 ± 10.9 ms, *p* > 0.10) or for low prices (426.1 ± 11.4 vs. 412.8 ± 11.6 ms, *p* > 0.10).

#### Response-locked ERPs

**Figure 5:**
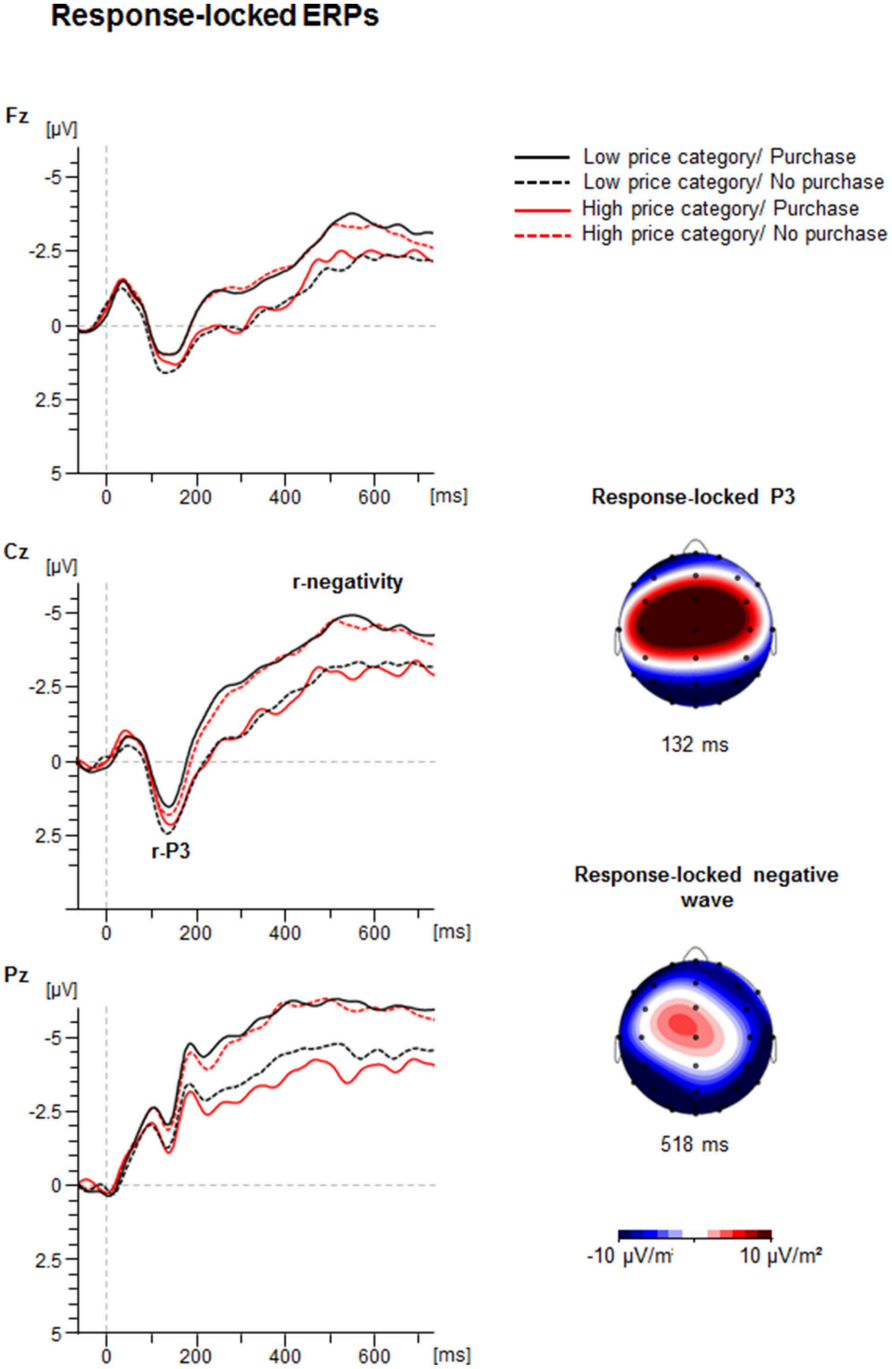
**Response-locked ERP-waveforms as a function of Price Category (low, high) and Purchase Decision (purchase, no purchase) at electrodes Fz, Cz, and Pz**. Dashed vertical line indicate response-onset (0 ms). The CSD maps illustrate a mean activity distribution for the response-locked P3 at 132 ms and for the response-locked negative ERPs at 518 ms.

Figure 5 illustrates the grand averages of the response-locked ERP-waveforms and the Current Density Maps (CSDs) of the analyzed ERPs. After the response a positivity with central maximum (r-P3) was seen. In addition, a slow negativity emerged with maximum at Pz, culminating at about 500 ms post-response.

##### Response-locked P3 (r-P3)

**Figure 6:**
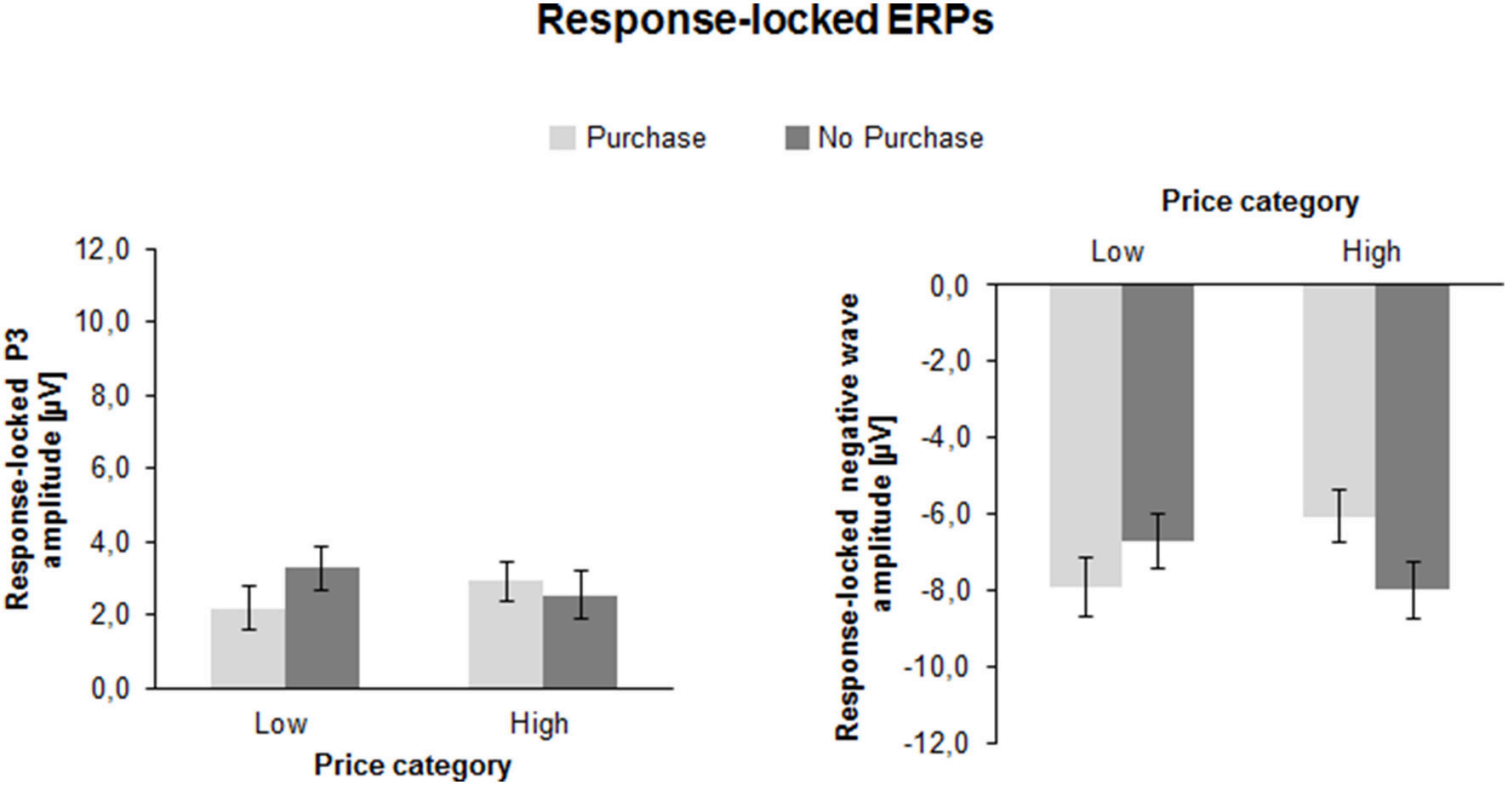
**Mean peak amplitudes of the response-locked ERPs (response-locked P3 and response-locked negative wave) as a function of Price Category (low, high), and Purchase Decision (purchase, no purchase)**. The error bars reflect SDs.

The ANOVA of the r-P3 peak amplitude yielded an interaction between Price and Decision as shown in Figure 6 [*F*_(1, 41)_ = 11.4, *p* < 0.01, η^2^ = 0.22]: if prices were low, the r-P3 was larger for negative (3.3 ± 0.6 μV) compared to positive decisions [2.2 ± 0.6 μV; *F*_(1, 41)_ = 8.1, *p* < 0.01, η^2^ = 0.16]. In contrast, for high prices, the amplitude was similar for positive (2.9 ± 0.5 μV) compared to negative decisions (2.5 ± 0.6; *p* > 0.10).

The r-P3 latency also yielded an interaction between Price and Decision [*F*_(1, 41)_ = 10.7, *p* < 0.01, η^2^ = 0.21]. For high prices, the latency was longer for purchase (151.9 ± 5.3 ms) compared to no purchase decisions [133.0 ± 5.3 ms; *F*_(1, 41)_ = 6.6, *p* < 0.05, η^2^ = 0.14], whereas for low prices no significant difference was found (130.0 ± 4.2 ms vs. 143.5 ± 5.8 ms; *p* > 0.05).

##### Response-locked negative ERP

The analysis of the response-locked negative going ERP amplitude revealed a substantial interaction between Price and Decision [*F*_(1, 41)_ = 25.7, *p* < 0.001, η^2^ = 0.39]. If prices were low, the ERP was more negative for purchase decision (−7.9 ± 0.8 μV) compared to no purchase decision [−6.7 ± 0.7 μV; *F*_(1, 41)_ = 6.1, *p* < 0.05, η^2^ = 0.13]. In contrast, for high prices, the response-locked negative ERP was more negative for no purchase decision (−8.0 ± 0.7 μV) compared to purchase decision [−6.1 ± 0.7; *F*_(1, 41)_ = 22.4, *p* < 0.001, η^2^ = 0.35]. This suggests larger post-response negativity for conform decisions compared to counter-conformity responses.

## Discussion

The aim of the present study was to elucidate effects of decision making in an economic context and to analyze their neuronal underpinnings. In particular, participants were asked to evaluate a product and its price and to decide either to buy or not to buy. The prices were either higher or lower relative to an average price estimated in a pilot study but the participants of the EEG-study did not know it. Moreover, faces with different emotional contents were assumed to influence the decisions. However, in contrast to our expectations and the previous results reported by Steffen et al. (2009), no effect of the emotional face valence on purchase decision and ERPs was observed. This was presumably due to a poor emotional expression of the used faces or alternatively facial expression has no relevance for the subsequent purchase decision to buy technical products. A further reason may be related to the procedure of the experiment. As the same facial expression was presented during 10 consecutive trials, the cognitive system may adapt and the effect would decay almost completely after a particular number of trials. We proved this possibility by analyzing the data of the first three trials after a switch of the emotional face valence to check for possible effects of adaptation but we still did not find any effects or interactions with this factor. A trial-by-trial manipulation using more pronounced face expressions and using shorter intervals between face presentation and decision would be presumably a better way to evoke an effect of emotional face expression on purchase decisions.

Expectedly, participants mostly decided to buy a product when it was cheap (about 70%) with decreasing acceptance rates (about 30%) as the offers became less attractive. The imbalance had consequences on reaction times as the rare decisions to buy an expensive product were longer than to buy a cheap product. The possible effects of the imbalance may also affect other outcomes as discussed below. More intriguing however, were effects of the counter-intuitive decision to buy a product for a higher than the average price or not to buy a product despite an attractive price. The behavioral data showed that the counter-conformity choices took significantly more time and the variability of the answer latency was substantially enhanced relative to the conform constellation, indicating more effort in dealing with conflicting choices. This cognitive and/or emotional effort in counter-conformity choices was reflected in a number of ERP findings. First of all, the interaction between price and readiness to buy a product was found in the fronto-central N2: the N2 was larger when participants decided to buy an expensive (vs. a cheap) product or not to buy a cheap (vs. expensive) product. As seen in Figure 4 the N2 amplitude did not differ between both conformity choices. A longer N2 latency was obtained for negative than for positive decisions. The conflict related N2 was usually investigated in context of incompatible response representations, which were defined by instruction. The novel approach in this study is that there is not an apparent conflict between an instructed correct or false response. As outlined above, a reference group estimated the range of appropriate vs. non-appropriate prices but the participants of the EEG sample defined themselves what represents the conflicting condition. The behavioral and EEG-results clearly showed that to purchase an expensive product or not to purchase a cheap product lead to more demanding, i.e., conflicting processing. The P3a and P3b results were similar to each other. The amplitudes were generally larger for conformity than for counter-conformity decisions. However, as seen in Figure 4, and in Chen et al. (2010a,b) this is most likely due to an overlap between the sustained negativities and the P3s, so true P3a and P3b effects are hard to extract from the target-locked averages. To obtain reliable effects at least for the P3b we relied more on the response-locked P3 (r-P3). The r-P3 amplitude was larger for counter-intuitive decisions, particularly in the low price category. The r-P3 latency was delayed for decisions to buy an expensive vs. cheap product. The opposite conflicting case not to buy a cheap product elicited merely a tendency for more delayed r-P3. In other words, assuming that the r-P3 reflects post-decisional processes (Nieuwenhuis et al., 2005; Verleger et al., 2005) counter-conformity responses tend to more intense and prolonged evaluation. Finally, as seen in Figure 5 there was a long lasting negative shift following the r-P3, the Contingent Negative Variation (CNV; Walter et al., 1964). The large difference between the conditions gave rise to a highly significant interaction between both factors price class and purchase decision. This interaction was due to larger CNV for conformity decisions, i.e., to buy a cheap product and to decline an expensive product. This is plausible as the preparation for the next trial can be larger if a simple and conformity decision has been made recently, fewer resources are needed for processing the response and resources can be fully devoted to preparation for the next trial (Falkenstein et al., 2003).

Taking together, the results regarding the N2 are clear-cut and support the hypothesis that this wave reflects resolution of conflicting information before a decision is completed. The role of the ACC, and pre-SMA in which the N2 is generated accords with this notion. These brain regions integrate cognitive and emotional inputs from different brain areas in order to reduce the available data to make a single decision (Paus, 2001; Botvinick, 2007; Nachev et al., 2008). The larger N2 amplitude in counter-conformity economic situations points to a larger cognitive or emotional effort to reach the response threshold and to complete the decision. This is also in line with the theory proposed recently by Shenhav et al. (2014), suggesting that ACC responds to decision difficulty. As mentioned above, Nieuwenhuis et al. (2005) suggested that the phasic response of the locus coeruleus underlying the P3b is driven by the activity of the decision process and is elicited as soon as this crosses the decision threshold. The pattern of P3a and P3b was equivalent and indicates smaller amplitudes for counter-conformity than conformity decisions. This pattern is roughly the opposite as found for the N2. A closer look at Figure 3 suggests that the later positivities are influenced by a negative shift which starts at about 250 ms and ends at about 600 ms. The pattern of the reduced late positive complex by a long-lasting negative shift (N500) is similar to that found by Chen et al. (2010a,b). In contrast, the r-P3 shows larger amplitudes for counter-conformity decisions after low prices, which suggests that it is less or not influenced by the negative shift. In summary, the results of the present study indicate that decision threshold can be affected by subjective relation between the price and attractiveness of the object. The larger the discrepancy between price and subjective value of a product the more demanding the decision due to higher decision threshold to buy a product. This decision takes more time and its evaluation is more difficult compared to non-discrepant situations. Future research should explore additional factors which may have impact on purchase threshold, for example past experience with particular products, personality factors, and cognitive biases, age or sociocultural background. Finally, it would be interesting to evaluate the effects of price vs. product order using the electrophysiological approach as qualitatively different expectations were established that may substantially affect purchase decisions.

## Limitations

There are also some limitations of the present study which have to be acknowledged.

First, it was an artificial situation in which participants were asked to virtually purchase a product without any incentive. Moreover, the same product was displayed multiple times with multiple prices. This induces a complex scenario in which subjects know that the same product may occur again for a lower price. There are multiple ways to approach this. One way would be to refer to the own estimate of the mean price and use that as a reference since the participants were not aware about the mean prices estimated in the pilot study. The alternative would be to sample within the experiment. One consequence of the latter interpretation of reference points might be that participants are in general less likely to make purchase decisions when they see the product for the first time.

Second, the outcome probability to buy a cheap product was 70% and to buy an expensive one was 30% which was associated with slower responses in the later compared to the earlier condition. The imbalance in decision frequency may also affect other parameters like the ERPs. The best example to illustrate frequency imbalance on the behavioral and electrophysiological data is the Oddball paradigm with infrequent targets that produce slower responses, more errors and larger P3b amplitudes compared to more frequent standard trials. However, in contrast to the Oddball paradigm with predefined targets and a fixed ratio of target and non-target trials the differences in the purchase rate in the present study occurred in a “natural” manner as cheap products are generally more frequently bought than expensive products. As the reaction times were longer for expensive than cheap products, it would merely suggest more effort invested in the decision process than when deciding about less expensive products. More importantly, the ERP pattern for low and high price category shows a consistent cross-over interaction between price category and purchase decision, whereas no main effect of price on the ERP component was found. This suggests no general imbalance between the two price conditions (at least in the ERP data) in contrast to the Oddball effect or frequency effects observed in other paradigms.

Third, we pooled the price levels in two price categories above and below the estimated averaged price. The reason for this was an unequal or too low number of trials in each price category that may substantially affect the quality of the ERPs.

Finally, the participants made a lot of decisions in a relatively short time (about 1 h) which may negatively affect the decision reliability at the end of the experiment. Thus, the generalizability and the transfer to real world behavior must be treated with caution.

## Conclusions

The present findings indicate that counter-conformity purchase decisions are related to longer and more variable responses. In the ERP they are accompanied by an increased N2, indexing enhanced processing of conflicting decisions. The N2 in counter-conformity decisions initiates a long-lasting negative shift, which reduces the amplitude of the late positive complex (P3a and P3b). Moreover it enhances and delays decision evaluation (r-P3), while it reduces the preparation for the subsequent trial (CNV). Hence, counterintuitive decisions have extended sequelae, which affect even the preparation for the next trial. We showed that the difficulty to make a counter-conformity decision elevates the response threshold and intensifies its evaluation.

This increased processing is not affected by facial expression, which was presumably too weak to evoke a strong emotional response. Further studies may induce stronger affective responses to evaluate their effects on the purchase decisions. Moreover, it is important to explore other factors like experience, age, or personality, which may influence the decision threshold.

## Author contributions

PG: Conception and design, data analysis, interpretation of the data, writing, and final approval. JD: Conception and design, programming, data acquisition, data analysis, interpretation of the data, writing, and final approval. JZ: Conception and design, interpretation of the data, writing, and final approval. MF: Conception and design, interpretation of the data, writing, and final approval.

## Funding

Funded by the Faculty of Mechanical Engineering, Ruhr University Bochum, Germany.

### Conflict of interest statement

The authors declare that the research was conducted in the absence of any commercial or financial relationships that could be construed as a potential conflict of interest.
